# A link between evolution and society fostering the UN sustainable development goals

**DOI:** 10.1111/eva.13728

**Published:** 2024-06-14

**Authors:** Luc De Meester, Ella Vázquez‐Domínguez, Rees Kassen, Félix Forest, Mauricio R. Bellon, Britt Koskella, Rosa A. Scherson, Licia Colli, Andrew P. Hendry, Keith A. Crandall, Daniel P. Faith, Craig J. Starger, R. Geeta, Hitoshi Araki, Ehsan M. Dulloo, Caroline Souffreau, Sibylle Schroer, Marc T. J. Johnson

**Affiliations:** ^1^ Leibniz Institute of Freshwater Ecology and Inland Fisheries (IGB) Berlin Germany; ^2^ Laboratory of Aquatic Ecology, Evolution and Conservation KU Leuven Leuven Belgium; ^3^ Institute of Biology Freie University Berlin Berlin Germany; ^4^ Berlin‐Brandenburg Institute of Advanced Biodiversity Research (BBIB) Berlin Germany; ^5^ Departamento de Ecología de la Biodiversidad, Instituto de Ecología, Universidad Nacional Autónoma de México Ciudad Universitaria Ciudad de México Mexico; ^6^ Conservation and Evolutionary Genetics Group Estación Biológica de Doñana (EBD‐CSIC) Sevilla Spain; ^7^ Department of Biology McGill University Montreal Quebec Canada; ^8^ Royal Botanic Gardens Kew, Richmond UK; ^9^ Comisión Nacional Para el Conocimiento y Uso de la Biodiversidad (CONABIO) México City Mexico; ^10^ Swette Center for Sustainable Food Systems Arizona State University Tempe Arizona USA; ^11^ Department of Integrative Biology University of California Berkeley California USA; ^12^ Laboratorio Evolución y Sistemática, Departamento de Silvicultura y Conservación de la Naturaleza Universidad de Chile Santiago Chile; ^13^ Dipartimento di Scienze Animali, Della Nutrizione e Degli Alimenti, BioDNA Centro di Ricerca Sulla Biodiversità e Sul DNA Antico, Facoltà di Scienze Agrarie, Alimentari e Ambientali Università Cattolica del Sacro Cuore Piacenza Italy; ^14^ Redpath Museum & Department of Biology McGill University Montreal Quebec Canada; ^15^ Department of Biostatistics and Bioinformatics George Washington University Washington DC USA; ^16^ Department of Invertebrate Zoology, US National Museum of Natural History Smithsonian Institution Washington DC USA; ^17^ University of Sydney Sydney New South Wales Australia; ^18^ School of Global Environmental Sustainability Colorado State University Fort Collins Colorado USA; ^19^ Department of Botany University of Delhi New Delhi India; ^20^ Research Faculty of Agriculture Hokkaido University Sapporo Japan; ^21^ Effective Genetic Resources Conservation and Use Alliance of Bioversity International and CIAT Rome Italy; ^22^ Department of Biology & Centre for Urban Environments University of Toronto Mississauga Mississauga Ontario Canada

**Keywords:** contemporary evolutionary change, ecosystem services, genetic diversity, past evolutionary change, phylogenetic diversity, present evolutionary change, sustainability science

## Abstract

Given the multitude of challenges Earth is facing, sustainability science is of key importance to our continued existence. Evolution is the fundamental biological process underlying the origin of all biodiversity. This phylogenetic diversity fosters the resilience of ecosystems to environmental change, and provides numerous resources to society, and options for the future. Genetic diversity within species is also key to the ability of populations to evolve and adapt to environmental change. Yet, the value of evolutionary processes and the consequences of their impairment have not generally been considered in sustainability research. We argue that biological evolution is important for sustainability and that the concepts, theory, data, and methodological approaches used in evolutionary biology can, in crucial ways, contribute to achieving the UN Sustainable Development Goals (SDGs). We discuss how evolutionary principles are relevant to understanding, maintaining, and improving Nature Contributions to People (NCP) and how they contribute to the SDGs. We highlight specific applications of evolution, evolutionary theory, and evolutionary biology's diverse toolbox, grouped into four major routes through which evolution and evolutionary insights can impact sustainability. We argue that information on both within‐species evolutionary potential and among‐species phylogenetic diversity is necessary to predict population, community, and ecosystem responses to global change and to make informed decisions on sustainable production, health, and well‐being. We provide examples of how evolutionary insights and the tools developed by evolutionary biology can not only inspire and enhance progress on the trajectory to sustainability, but also highlight some obstacles that hitherto seem to have impeded an efficient uptake of evolutionary insights in sustainability research and actions to sustain SDGs. We call for enhanced collaboration between sustainability science and evolutionary biology to understand how integrating these disciplines can help achieve the sustainable future envisioned by the UN SDGs.

## 1 INTRODUCTION

Given the multitude of challenges Earth is facing, sustainability science is of key importance to our continued existence. Sustainability science studies the interactions between natural and social systems, and how those interactions affect the challenge of sustainability: meeting the needs of present and future generations while substantially increasing human well‐being and conserving the planet's life support systems (Clark & Dickson, 2003). Ecosystem services and the broader framework of “Nature's Contributions to People” (NCP) are well‐established concepts that are core to sustainability science (Dasgupta et al., 2022; Díaz et al., 2018). Evolution—the change in gene or allele frequencies over time within a population (Freeman & Herron, 2007)—is the core process generating the biological diversity that supports all aspects of ecosystem function and NCP (Des Roches, Pendleton, et al., 2021a; Díaz et al., 2018; Flynn et al., 2011; Isbell et al., 2011; Lefcheck et al., 2015) but is not usually considered directly in sustainability science or policy (Messerli et al., 2019). Thus, the value of evolutionary processes and the consequences of their impairment have not generally and explicitly been considered in our efforts to achieve sustainability (Carroll et al., 2014; Faith et al., 2010; Vázquez‐Domínguez et al., 2024).

In this Perspective, we articulate the contributions biological evolution makes to sustainability and show how the concepts, theory, data, and methodological approaches used in evolutionary biology can contribute to achieving the UN Sustainable Development Goals (SDGs). We first discuss how evolutionary principles are relevant to understanding, maintaining, and improving NCP, including ecosystem services, and the SDGs. Second, we develop a framework showing how evolutionary biology supports sustainability science by clarifying how evolutionary principles and tools are used to make informed decisions in broad target fields (e.g., health and food production). Third, we briefly highlight barriers to a better recognition of evolutionary biology in sustainability research. We end by making a plea for evolutionary biologists to be more sensitive to the implications of their work with respect to trajectories for sustainability, and, in turn, for the community of sustainability research to be more open to incorporate evolutionary insights and tools in their research and development of scenarios.

## 2 NATURE'S CONTRIBUTIONS TO PEOPLE AND THE SUSTAINABLE DEVELOPMENT GOALS

Nature's Contributions to People (NCP) are all the contributions, both positive and negative, of living nature (diversity of organisms, ecosystems, and their associated ecological and evolutionary processes) to people's quality of life (Díaz et al., 2018). The importance of NCP to sustainability is well‐recognized. NCP offers a portfolio of solutions, or option values, to sustainability challenges such as foods with improved nutrition (SDG 2), drugs to combat disease (SDG 3), and sources of alternative renewable energy (e.g., algae for biofuels—SDG 7). The taxonomic diversity NCP rests on also provides essential services such as seed dispersal, pollination, and degradation of pollutants that provide healthy, resilient ecosystems to support socio‐economic well‐being (SDG 6, SDG 12, SDG 13).

**FIGURE 1:**
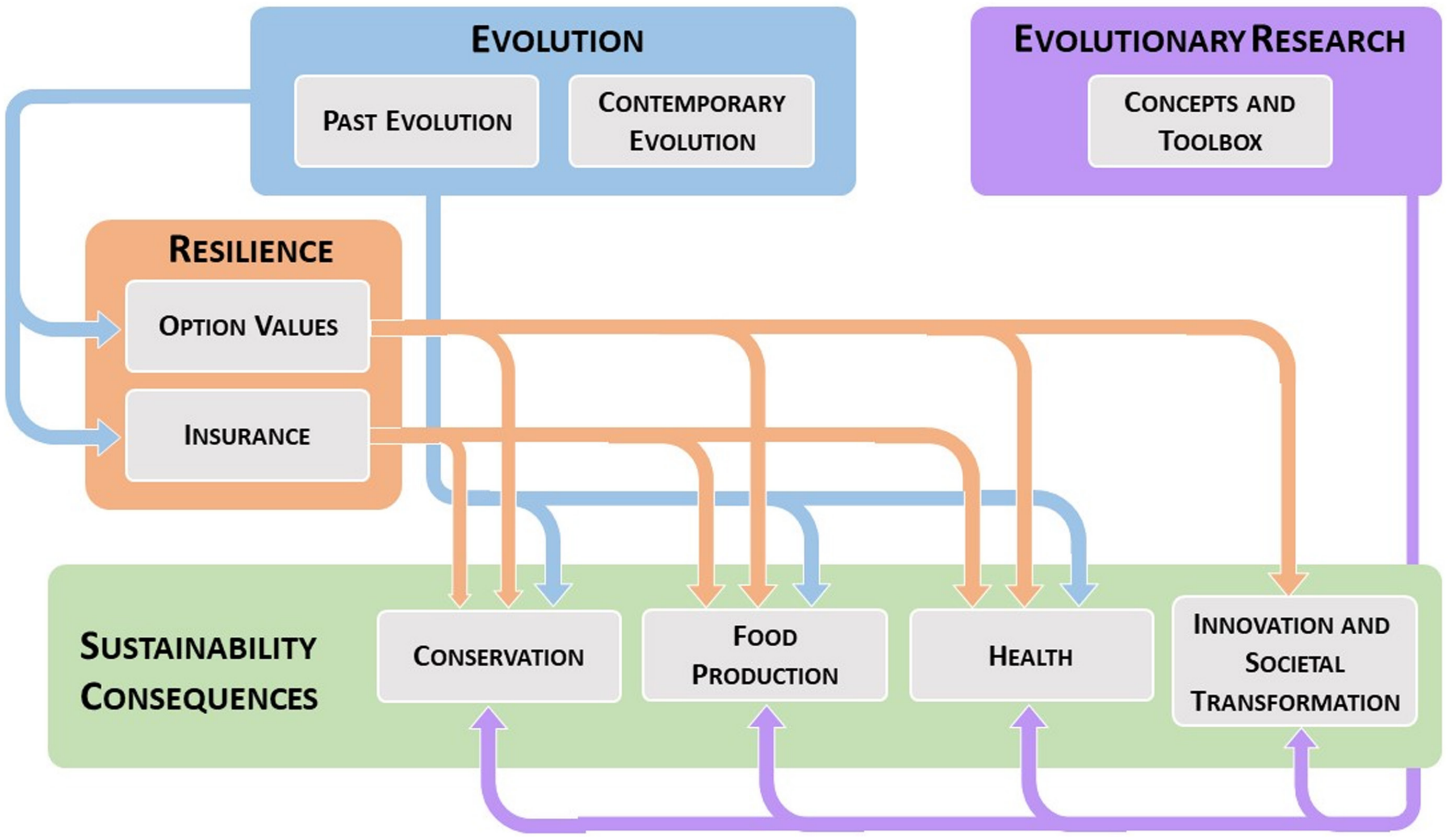
Schematic diagram depicting the contribution of past (i.e., phylogenetic diversity) and contemporary evolution, and of evolutionary biology's toolbox, to four important aspects of sustainability: (1) conservation, (2) food production and security, (3) health, and (4) innovation and societal transformation. The contribution of evolution can be direct or through the insurance function of genetic and phylogenetic diversity and option values, both supporting the resiliece of ecosystem services and the maintenance of options on possible future uses and benefits of biodiversity, in the face of human‐induced environmental change.

Ample evidence has accumulated that evolution provides crucial benefits to human well‐being along many dimensions related to NCP. One compelling and obvious example is that evolutionary processes are ultimately responsible for the generation and maintenance of the diversity of domestic and wild foods that sustain our species. Other examples are highlighted in Table 1, grouped according to the SDG to which they are most closely aligned. The term “evosystem services” was coined to capture the important contribution of evolution to NCP (Faith et al., 2010). In part, this contribution is linked to the capacity of evolutionary processes to buffer environmental change (Dakos et al., 2019; Urban et al., 2020), which enhances the resilience of ecosystems and their associated services to society (Hughes et al., 2008). In this context, evolutionary trait change can, for example, modulate ecosystem tipping points by delaying or preventing the collapse of an ecosystem (Chaparro Pedraza et al., 2021; Dakos et al., 2019). Both among‐species phylogenetic diversity that captures the evolutionary history of species (macroevolution), and within‐species genetic diversity that fuels contemporary evolution (microevolution), contribute to the insurance function of biodiversity, safeguarding the functioning of ecosystems and thus ecosystem services in the face of environmental change (Figure 1). The impact of genetic variation on such resilience reflects the fact that genetic diversity can stabilize populations and communities by allowing adaptive responses to altered environments (Bell, 2017; Díaz et al., 2013). In addition, both phylogenetic diversity and contemporary evolution contribute to options for the future (Figure 1; see IPBES NCP18 on “Maintenance of options”; Díaz et al., 2019). Option values are the value of maintaining variation within species and across phylogenies to provide possible future uses and benefits (Díaz et al., 2018). Evolutionary option values result from particular species and evolutionary lineages harboring unique features that have emerged as the result of billions of years of evolution and might prove to be important to future human societies (Molina‐Venegas, 2021). Therefore, NCP are strongly affected by evolutionary processes, making the understanding of those processes a key component as we strive towards a sustainable society.

**TABLE 1:** Evolutionary contributions to the UN Sustainable Development Goals (SDGs) with example references.

SDG	Tar‐gets	A	NDA	Evolutionary contribution	Example reference
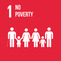	7	3	3	While evolutionary insights are not directly applicable to this SDG, evolution and evolutionary biology are indirectly relevant through the use of evolutionary insights and the evolutionary toolbox to increase food, feed, fiber and energy production, and the production of valuable biochemicals, in a sustainable way. Evolutionary biology can help address underlying factors that contribute to poverty, such as food security, health, and nature's benefits to people. The use of evolutionary insights to facilitate societal transitions can also contribute to this SDG. Phylogenetic and genetic diversity increase the resilience of ecosystems to global change, directly relevant to Target 1.5. (“…reduce their exposure and vulnerability to climate‐related extreme events and other economic, social and environmental shocks…”). The importance of ownership of local seeds, breeds and genetic resources is directly relevant to Target 1.4 (“Ensure that all men and women, …, have equal rights economic resources, as well as access to basic services, ownership and control over land and other forms of property, inheritance, natural resources …”). Assistance and development programs should consider evolutionary thinking when designing and implementing their food security, global health, and environment programs.	Asokan et al. (2017); Cleveland and Murray (1997); Gould et al. (2018); Lewis and Steinmo (2012); Mendelsohn (2003); Nielsen et al. (2012); Nill and Kemp (2009)
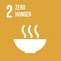	8	7	1	Direct applications of evolution, evolutionary insights and the evolutionary toolbox to increase food production in a sustainable way. Phylogenetic and genetic diversity increase the resilience of production ecosystems to global change. Ownership of local seeds, breeds and genetic resources is directly relevant to Target 2.3 (“Double agricultural productivity and income of small‐scale food producers”). The importance of genetic variation, evolutionary distinct and locally adapted landraces and breeds that allow people to live in diverse environments in the face of climate change is directly relevant to Target 2.4 (“Ensure sustainable food production systems and implement resilient agricultural practices that increase productivity and production, that help maintain ecosystems, that strengthen capacity for adaptation to climate change, extreme weather, drought, flooding and other disasters and that progressively improve land and soil quality”). With respect to Target 2.5 (“Maintain the genetic diversity of seeds, cultivated plants and farmed and domesticated animals and their related wild species, …”), evolutionary concepts inform us on the need to go beyond seed banks and cryopreservation, and also engage in the maintenance of active populations of many landraces and local breeds under a wide range of circumstances to sustain evolution and the maintenance of genetic variation. Increased efficiency to improve domestic species and crops by marker‐assisted selection	Ahrens et al. (2020); Andersson and Purugganan (2022); Bellon et al. (2017); Dana et al. (2010); Denholm et al. (2002); Eş et al. (2019); Fleury et al. (2010); Gassmann et al. (2011); Hasan et al. (2021); Köhler‐Rollefson et al. (2009); Kulus (2019); Marco‐Jiménez et al. (2018); McCart et al. (2005); Mercer et al. (2008); Nielsen et al. (2012); Ruto et al. (2007); Scherf and Pilling (2015); van Zonneveld et al. (2012); Wakchaure et al. (2015); Welch et al. (2013)
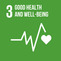	13	4	7	Many direct applications of evolution and evolutionary insights. Amongst others: Recognize the emerging threat of Multiple Antibiotic Resistance and use evolutionary inspired approaches to reduce its occurrence and impact. Apply evolutionary principles on epidemiology and to improve predictions of changes in virulence of pathogens through time. Use evolutionary theory to improve predictions on emerging diseases. Apply evolutionary principles to prevent development of resistance of vectors and pathogens to pesticides (e.g. insecticide resistance in mosquitoes; resistance to fungicides). Using evolutionary theory to enhance the establishment and spread of the intracellular parasite Wolbachia to suppress disease vectors. Apply evolutionary principles on bacteriophage treatments. Consider evolutionary responses of vectors and pathogens to climate change and land use change. Apply evolutionary theory and insights to the development of effective vaccines and drugs, especially antibiotics. Effectiveness also involves delivery and dosing. Importance of evolutionary insights in developing effective treatments for cancer. Importance of evolutionary insights in understanding diseases linked to the gut microbiome. Development of population genetics and other tools for detection of health risks. Using molecular tools and phylogenies to reconstruct the history of disease outbreaks. Relevance of biodiversity, including phylogenetic diversity, on mental well‐being. Understand the development of chronic infections Potential to offer more diversified diets	Anthony et al. (2015); Baym et al., 2016a, (2016b); Dettman and Kassen (2021); Flores and O'Neill (2018); Greaves and Maley (2012); Harvey and Holmes (2022); Hoffmann et al. (2011); Innes et al. (2022); Ling et al. (2015); MacLean and San Millan (2019); Megharaj et al. (2011); Meng et al. (2022); Merlo et al. (2006); Nesse and Stearns (2008); Ou et al. (2013); Rašić et al. (2014); Read and Mackinnon (2007); Ross et al. (2019); Ross and Hoffmann (2021); Sánchez‐Rivera and Jacks (2015); Schloissnig et al. (2013); Schrag and Wiener (1995); Thomas et al. (2020); Vicens and Posada (2018); Willett et al. (2019); Wölfl et al. (2022); Zhan et al. (2019)
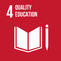	10		2	Good quality education should involve evolutionary theory, given its importance in sustainability issues and to properly interpret information on genetics and evolutionary dynamics such as the evolution of antibiotic resistance. Better education in evolution will also improve understanding of consequences of mismanagement of biodiversity and the non‐thoughtful applications of concepts and techniques that might interfere with biodiversity conservation.	De Salle and Perkins (2016); Sinatra et al. (2008)
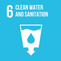	8	4	4	Multiple direct applications of evolutionary insights and tools, including: Evolution of resistance and tolerance to toxins and pollutants. Evolutionary aspects of bioremediation. Applying the evolutionary toolbox to detect and isolate bacteria with specific bioremediation relevant properties. The importance of phylogenetic diversity for water purification. Evolution‐mediated resilience leading to maintenance of clear‐water conditions in urban ponds and lakes Less water pollution through a reduced use of agrochemicals and nutrients inspired by selection or modification of natural disease resistance.	Des Roches, Brans, et al. (2021b); Lambert and Donihue (2020); Pespeni et al. (2013); Timmis and Pieper (1999)
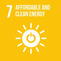	5	1	4	Direct applications of evolution and the evolutionary toolbox include: Exploring phylogenetic diversity and apply selection to increase the efficiency of algal strains and plants for biofuel production Biomimetic approaches (e.g. evolution inspiring more efficient solar cell design). The large‐scale land use linked to biofuel production may also necessitate conservation genetic studies on the consequences for evolutionary potential and phylogenetic diversity of natural habitats.	Stephenson et al. (2011); Wang et al. (2013)
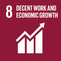	12	0	9	There are indirect links, in the sense that evolutionary research, biotechnology, applications of the evolutionary toolbox, Darwinian medicine, biotechnology, biomimicry, developing evolutionary algorithms, etc., can generate a diverse set of jobs and yield economic and societal benefit, also on the long term if applied in a setting that focuses on sustainability.	Collins et al. (2016)
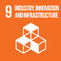	8	2	3	The many applications of evolutionary insights, evolutionary algorithms, biomimetics and the evolutionary toolbox yield opportunities for new industries and spin‐offs. Evolutionary algorithms can be used to solve contemporary design and process challenges. Applications of Evolutionary Stable Strategies in economics.	Bai et al. (2022); Bar‐Cohen (2006); Deng et al. (2021); Eiben and Smith (2003); Friedman (1998); Safarzyńska et al. (2012); Slowik and Kwasnicka (2020); Wang et al. (2013)
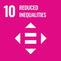	10		4	Better understanding of evolution reduces discrimination, amongst others by fostering a better universal understanding of shared human ancestries. Evolutionary insights and algorithms can be used to foster change. Determine drivers for linguistic diversity	Gavin et al. (2013); Lewis and Steinmo (2012)
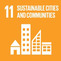	10	2	4	Evolutionary algorithms can be used to optimize urban transport planning. Biomimetics of social insect community organisation. Taking urban evolution and the impact of urbanisation on population connectivity and the distribution of genetic variation across the landscape into account in urban planning.	Alberti (2015); Biek and Real (2010); Des Roches, Brans, et al. (2021b); Johnson and Munshi‐South (2017); Lambert and Donihue (2020); Miles et al. (2021); Rivkin et al. (2019); Santangelo et al. (2022); Szulkin et al. (2020)
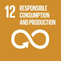	11	2	7	Evolutionary approaches to facilitate regime shifts in attitudes. Selection for higher energy foods and plant varieties that can grow on degraded lands or use less water. Selection for higher pathogen resistance or tolerance to grazers so as to increase sustainability of food production. Selection of more efficient livestock breeds that combine high yields, disease resistance, climate change resilience with reduced GHG emissions and use of antibiotics. Optimizing the use and regulation of pesticides	Denholm et al. (2002); Eş et al. (2019); Fleury et al. (2010); Hasan et al. (2021); Kreiner and Booker (2023); Lewis and Steinmo (2012); Mercer et al. (2008); Scott et al. (2018); Wakchaure et al. (2015)
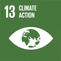	5	2	2	Direct applications through the implementation of evolution‐inspired modelling on climate and climate adaptation, and the use of evolutionary insights to predict how evolution impacts responses of populations, communities and ecosystems to climate change. Link biodiversity hotspots and species distribution modelling to present and future areas of evolutionary interest under different climate change scenarios Increase long‐term genetic diversity to ensure diversification of food systems and food security in the future	Bay et al. (2018); Bjarklev et al. (2019); Cai et al. (2021); Geisendorf (2018); Jump and Peñuelas (2005); Qian et al. (2023); Rodriguez et al. (2022); Sgrò et al. (2011); Urban et al. (2012, 2016); Voskamp et al. (2022)
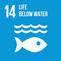	10	4	6	Many applications, including: Conservation genetics and evolutionary insights informing management decisions. Importance of phylogenetic and genetic diversity for resilience of ecosystems. Importance of evolutionary insights in sustainable fish stock management. Using the molecular toolbox for monitoring sustainability of fishing or success of marine reserves. Importance of phylogenetic diversity as option values in freshwater and marine systems (biomimetic approaches, selection for specific features).	Ahrens et al. (2020); Conover and Munch (2002); Crandall et al. (2000); Des Roches, Brans, et al. (2021b); Fraser and Bernatchez (2001); Lambert and Donihue (2020); Nielsen et al. (2012); Olsen et al. (2004); Piaggio et al. (2017); Sgrò et al. (2011)
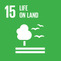	12	9	1	Many applications, including: Conservation genetics and evolutionary insights informing management decisions. Importance of phylogenetic and genetic diversity for resilience of ecosystems. Importance of evolutionary insights in forest management. Using the molecular toolbox for monitoring sustainability of wood harvesting or success of protected areas. Importance of phylogenetic diversity as option values in forests and wetlands (biomimetic approaches, selection for specific features).	Brooks et al. (2015); Crandall et al. (2000); Des Roches, Brans, et al. (2021b); Fraser and Bernatchez (2001); Harrisson et al. (2014); Iwona et al. (2018); Johnson et al. (2010); Lambert and Donihue (2020); Sgrò et al. (2011)
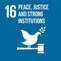	12		1	Better understanding of evolution reduces prejudice. Evolutionary algorithms to facilitate peaceful change and transition. Evolution‐inspired structure of institutions.	Kite & Whitley (2016); Lewis and Steinmo (2012)
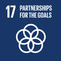	19			Evolutionary approaches to facilitate regime shifts in attitudes and reform institutes.	Lewis & Steinmo (2012)

*Note*: The % of the SDG targets to which evolution can contribute is indicated as A = Applicable, NDA = Not directly applicable, but relevant in more indirect ways. The total number of targets as identified in the United Nations Department of Economic and Social Affairs (https://sdgs.un.org/goals) and the percentage of these for which evolution or evolutionary insights are relevant is indicated in Figure 2.

Discussions of NCP and option values emphasize the positive contributions of evolutionary processes to generating the biological diversity on which sustainability rests. It is equally important to remember that evolutionary processes can also create challenges for society and ecosystem sustainability, such as when microorganisms like viruses and bacteria adapt to evade strategies designed to prevent disease transmission. We need to look no further than our collective experience managing variants of concern during COVID‐19, for example. The same evolutionary processes that underpin positive contributions of biodiversity to sustainability also need to be considered when trying to avoid evolutionary “disservices” impacting ecosystem and human health (Gould et al., 2018; MacLean & San Millan, 2019).

**FIGURE 2:**
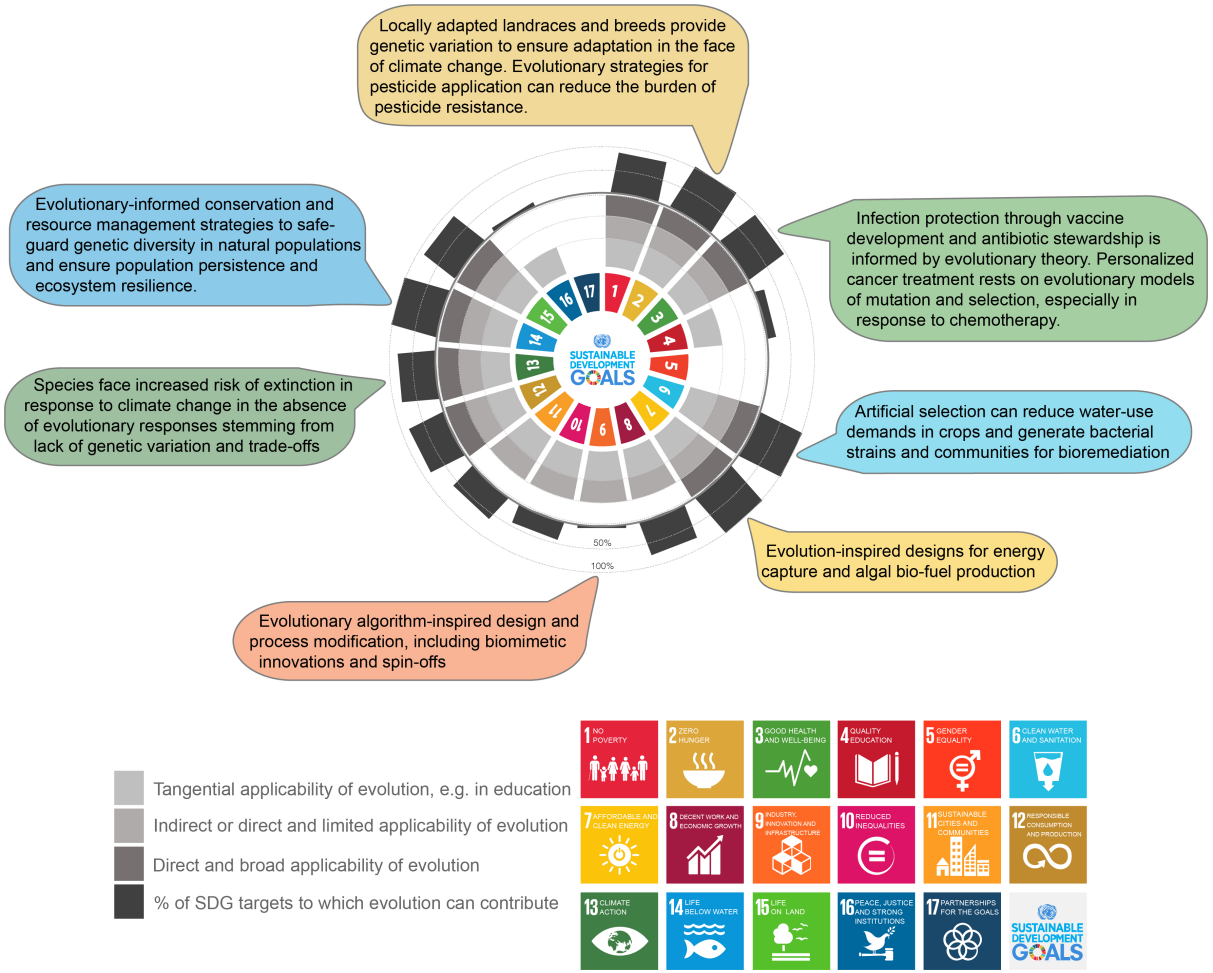
Schematic representation of evolutionary contributions to the UN Sustainable Development Goals, with indication of the type of applicability (tangential, indirect and limited, direct, and broad). The percentage (%) of the SDG targets to which evolution can contribute is indicated. See also Tables 1 and 2 for studies illustrating the different types of applications.

In light of evolution's ability to contribute both positively and negatively to our collective well‐being, we suggest that considering evolutionary concepts, theory, insights, data, and tools are necessary to achieve many of the SDGs. To make this more concrete, we have collated examples from the literature highlighting evolution's contributions to each of the 17 SDGs in Table 1 and summarized this exercise schematically in Figure 2. For some SDGs like SDG 14 (Life below water) and SDG 15 (Life on land), it should be self‐evident that evolutionary processes contribute to sustainability by generating among‐ and within‐species diversity, contributing to ecological productivity and population resilience in the face of environmental change and disturbance (Adolf et al., 2020; Bell, 2017). Evolution can also make important contributions to other SDGs that might at first glance be less obvious or indirect, such as when small‐scale farming practices lead to the development of locally adapted crop varieties and so contribute to achieving SDG 2 (Zero hunger), or the use of evolutionary principles in algorithm design in engineering for SDG 9 (Innovations, Industry, and Infrastructure). Table 2 provides further examples of the application of evolutionary principles and tools that are used to achieve the SDGs. What should be obvious from the literature reviewed here is that evolutionary processes and tools are already widely used in the context of sustainability, even if they are not always recognized as such.

**TABLE 2:** Applications of the evolutionary toolbox.

Techniques/statistical tools	Developed to (examples)	Applications (reference; SDG)
CRISPR‐Cas9	Gene editing mechanism discovered by studying the naturally evolved defense responses in bacteria and archaea against viruses (Horvath & Barrangou, 2010, Ran et al., 2013)	Genome editing (Wang et al., 2016; SDG 9), Food production (Eş et al., 2019; SDG 2, SDG 12) Human health (Sánchez‐Rivera & Jacks, 2015; Zhan et al., 2019; SDG 3) Conservation (Desalle & Amato, 2017; Hsu et al., 2014; Piaggio et al., 2017; SDG 14, SDG 15)
Marker‐assisted selection Genomic variants in local landraces and breeds	Improve domestic species and crops (Hasan et al., 2021; Wakchaure et al., 2015) Select of adaptations to extreme environmental conditions	Introduce in the genomes of commercial populations, thus conferring adaptive traits without altering their existing beneficial adaptations (Hasan et al., 2021; Wakchaure et al., 2015; SDG 2, SDG 12)
Genomic tools	Assess fish stock identity	Monitoring adherence to fisheries policies (Nielsen et al., 2012; SDG 1, SDG 2, SDG 14)
Selective breeding	Enrich the original genome of individuals of species threated with extinction	Reintroduction, traslocation (Miller et al., 2017; SDG 14, SDG 15)
DNA metabarcoding	Genomic sequencing of soil, water, ice and air samples	Ecosystem and human health, among others (Chen et al., 2012; Nguyen et al., 2020; SDG 3, SDG 6, SDG 14, SDG 15)
Diverse molecular and phylogenetic tools	Genetically characterize microbiome, virus, bacteria	Trace pathogen origins, outbreaks; screen for disease (De Salle & Perkins, 2016; SDG 3)
Evolutionary algorithms	Identify evolution pathways	Broad range of engineering problems (Bai et al., 2022; Deng et al., 2021; Slowik & Kwasnicka, 2020) Example: antennas for the NASA Space Technology 5 telescope (Eiben & Smith, 2003) (SDG 7, SDG 9, SDG 11)
Evolutionary game theory	Evaluate vaccination behavior of individuals	Explore effectiveness of vaccination strategies (Meng et al., 2022) Personalized cancer treatments (Wölfl et al., 2022) (SDG 3)

*Note*: Brief account of examples of techniques and statistical tools developed to study evolution and their current/potential applications to inform sustainable policies (e.g., Sustainable Developmental Goals ‐ SDGs).

## 3 HOW EVOLUTIONARY BIOLOGY SUPPORTS SUSTAINABILITY SCIENCE

We here discuss in more detail how evolutionary thinking and knowledge can help tackle societal challenges relevant to different dimensions of sustainability and the SDGs, including urbanization, fisheries management, health, food production, and conservation of natural resources.

### 3.1 Fisheries management

Intensive fisheries induce the evolution of reproductive and migration timing, as well as time and size of maturation, which may delay the recovery of commercial fisheries (Conover & Munch, 2002; Olsen et al., 2004) (SDG 1, SDG 2, SDG 3, SDG 14). Fisheries management that incorporates evolutionary thinking can substantially improve sustainable harvests and recovery from fisheries collapses (Ahrens et al., 2020; Laugen et al., 2014; Matsumura et al., 2011) (SDG 1, SDG 2, SDG 3, SDG 14). Understanding which species can and cannot evolutionarily adapt to human‐induced change, including pollution and ocean acidification (Reid et al., 2016; Schlüter et al., 2014), as well as the rate of these adaptations, will be key to predicting how marine populations and communities will change in the future, impacting the effective management of many commercially important fisheries.

### 3.2 Urban evolution

Urban environments create new kinds of selection pressures on populations and species that can have dramatic impacts on plant and animal life as well as ecosystem services (Brans et al., 2022). Examples include changes in dispersal modes in plants, altered morphology and decreased migratory behavior in birds, different thermal adaptation in butterflies, water fleas, and plants, more exploratory and less shy personality types in various animals, altered responses to infectious and zoonotic diseases, and increased pollution tolerance, to name a few (Biek & Real, 2010; Johnson & Munshi‐South, 2017; Miles et al., 2021; Santangelo et al., 2022; Szulkin et al., 2020). Urban‐induced evolution can be aligned with sustainability goals, when it contributes to safeguarding crucial ecosystem services like pollination, control of pest species, and maintenance of clear‐water conditions in city ponds and lakes (Des Roches, Brans, et al., 2021b; Lambert & Donihue, 2020; Vázquez‐Domínguez et al., 2024) (SDG 6, SDG 11, SDG 14, SDG 15). It can also create new problems, such as when urban pollution induces de novo mutations in organisms (Somers et al., 2004; Yauk & Quinn, 1996) linked to diseases such as cancer. From a policy perspective, the spectrum of evolutionary responses to urbanization can thus range from positive to negative, creating tensions around the functioning of urban ecosystems, the management of wildlife in urban centers, and the contribution of pollution to health that need to be weighed in decision‐making.

### 3.3 Sustainable agriculture

Intensive agricultural practices have diverse consequences, including widespread pollution and eutrophication (Delabre et al., 2021; Fu et al., 2022; Glaros et al., 2022; Moss, 2008), disrupting species interactions (Knipler et al., 2022; Porter & Sachs, 2020; Stephens et al., 2022), and creating strong selection pressures on pathogens, parasites, commensals, insects, weeds, and their consumers (Baidya & Bagchi, 2022; Madeira et al., 2022; Nova et al., 2022; Ortiz et al., 2021) (SDG 9, SDG 12). The evolution of herbicide resistance in weeds and pesticide resistance in insects are examples of a growing problem that can only be tackled by incorporating evolutionary dynamics and insights into optimizing the use and regulation of pesticides (Gould et al., 2018; Kreiner & Booker, 2023).

The conservation of a diversity of crops and livestock reproductive material (e.g., semen doses, gametes, embryos) in gene banks constitute “snap‐shots” of the genetic diversity present at the time of collection. This type of conservation, although important for preserving the genetic variation that supports continued innovation (SDG 9), is insufficient on its own to ensure the long‐term viability of breeds and varieties (Bellon et al., 2017). Hence, maintaining the evolutionary processes and genetic diversity of cultivated plants and farmed animals is essential for ensuring their capacity for adaptation, as well as for sustainable food production systems (Andersson & Purugganan, 2022; Bernatchez et al., 2017; Scheben et al., 2016; Scherf & Pilling, 2015) (SDG 2).

In contrast to agri‐business practices that focus on intensive, large‐scale cultivation of one or a few highly productive varieties, evolution under domestication is often driven by farmers growing landraces and local breeds (Meyer & Purugganan, 2013). In one example, many small‐scale farmers grow a broad diversity of native landraces of maize in varying environments in Mexico, its center of domestication and diversity (Bellon et al., 2018). Estimates based on the different individual plants that are subjected to evolution under domestication each season suggest that 5.24 × 10^8^ mother plants contribute standing genetic diversity and rare alleles to the next generation. Small‐scale cultivation and selection of local landraces and breeds can thus increase the total number of adaptive mutations available for selection under domestication (Bellon et al., 2018). It also provides invaluable genetic material for local adaptation and resiliency that can enhance food security in the face of threats from climate change, including novel pathogens, droughts, and flooding (Vigouroux et al., 2011). Ensuring that these farmers maintain the right to save and share seeds and breeding animals is fundamental to enabling evolution under domestication (SDG 12), contributing to breaking the cycle of poverty (SDG 1), and empowering individuals to access sufficient food (SDG 2). Genetic diversity in wild relatives can be used to improve disease and stress resistance in crops and domestic animals and thus protect yields in a more reliable and sustainable fashion (Milliken et al., 2021; Mundt, 2002; Zhang et al., 2017; Zhu et al., 2000) (SDG 12).

### 3.4 Health and well‐being

Evolution contributes to healthcare and medicine (Garnås, 2022; Natterson‐Horowitz et al., 2023), both in terms of providing insight into why symptoms and diseases occur and by offering new therapeutic options (Gluckman et al., 2016) (SDG 3). Evolutionary theory is crucial to predicting epidemiological dynamics, including disease outbreaks (Harvey & Holmes, 2022; Schrag & Wiener, 1995) and changes to both transmission dynamics and virulence, as exemplified by the repeated emergence of COVID‐19 variants of concern (Otto et al., 2021). Phylogenomic analyses, which use genomic sequence information to reconstruct the evolutionary history of pathogens and to build transmission networks, are used to identify virulence determinants and, when combined with epidemiological information and experimental studies of virus virulence and fitness, guide public health interventions and treatment options, including vaccination (Meng et al., 2022; Read & Mackinnon, 2007) (SDG 3). These kinds of evolutionary predictions are especially important in a world in which transmission dynamics are changing rapidly due to global transportation, climate change, and habitat loss (Lebarbenchon et al., 2008), which collectively lead to the emergence or re‐emergence of infectious disease.

Evolutionary insights have also been increasingly applied to better understand the development of chronic infections (Dettman & Kassen, 2021) and of cancer (Anand et al., 2022; Dsouza et al., 2022; Thomas et al., 2020), such as through personalized cancer treatments developed using evolutionary game theory (Wölfl et al., 2022). Gene drives, an allele of a diploid gene that is inherited more than 50% of the time (that is, more than by random chance), offer the potential to reduce the prevalence of vector‐borne diseases, crop pests, and non‐native invasive species (Bier, 2022). Examples of these gene‐drive systems aimed at controlling disease vectors include the addition of vector competence genes with the Cas9–gRNA (CRISPR) construct, resulting in virus‐resistant offspring (Gantz et al., 2015). Sterile females have been obtained via the creation of a gRNA targeting female fertility genes (Hammond et al., 2016), as well as a reduction in female offspring with a gRNA targeting X chromosome‐specific sequences (Flores & O'Neill, 2018; Ross et al., 2019; Ross & Hoffmann, 2021). Gene drives could also reduce the use of pesticides in agriculture (Busby et al., 2017; Scott et al., 2018).

A very promising avenue for using evolutionary principles for sustainability associated with human health involves our relationship with microbes. Antibiotic resistance (i.e., selection for antibiotic‐resistant bacterial strains) resulting from rapid evolution by microbial populations in response to treatment (e.g., antibiotics for bacterial infections, vaccines for viruses; Murray et al., 2022; Pulingam et al., 2022) pose a serious threat to humans, animal populations, and ecosystems (Larsson & Flach, 2022; Magouras et al., 2017). In other words, the emergence of resistance is an evolutionary phenomenon; preventing resistance, or managing its spread once it evolves, thus demands an evolution‐informed response (Leale & Kassen, 2018). Compelling examples where evolutionary principles have been used to combat resistance include the use of multiple selective forces, such as those imposed by bacteriophage viruses or drug combinations on pathogen populations to prevent or slow the spread of resistant variants (Burrowes et al., 2011; Chan et al., 2016; Chow et al., 1993; Hatfull et al., 2022). Finally, the link between gut microbiomes and health is an important research area (Shreiner et al., 2015), including how microbiomes are shaped by and influence evolutionary dynamics (Zhao et al., 2019). Phylogenomic diversity and abundance‐based metrics, as well as phylogenetic methods for community comparisons (Chen et al., 2012; Matsen 4th, 2015; Risely et al., 2021), are currently used to investigate links between gut microbial diversity and human and animal health. Low microbial phylogenetic diversity has been associated with several diseases, including obesity, inflammatory bowel disease, and psychiatric disorders (Stanislawski et al., 2019); also, diverse studies have shown that gut microbiota influences health, immune response, behavior, and stress in farm animals (Chen et al., 2021).

### 3.5 Conservation

Evolutionary principles and processes have become increasingly embedded in conservation biology (Crandall et al., 2000; Mimura et al., 2017). Evolutionary biology permeates conservation through prioritization of taxa for conservation management, the focus on Evolutionary Significant Units (ESUs) (Fraser & Bernatchez, 2001), understanding the role of natural selection, hybridization, and admixture among populations of conservation concern (von Holdt et al., 2018), studies quantifying inbreeding and genetic drift as drivers of loss of genetic diversity in small populations (so‐called “genetic erosion”), and designing management plans to restore genetic variation (Johnson et al., 2010) (SDG 14, SDG 15). The use of modern genomic data has made it possible to evaluate current and future risks of inbreeding, and to quantify the adaptive potential and, thus, the resilience of species to environmental change (Dauphin et al., 2023). Although uptake of these approaches was initially slow due to cost constraints, conservation organizations are increasingly appreciating that without incorporating evolutionary thinking into their management plans, “future proofing” threatened and endangered populations to the Anthropocene will be difficult, if not impossible. Evolutionary thinking and tools can help identify areas that harbor a larger‐than‐expected evolutionary potential (Faith, 2018), which can function as evolutionary refugia to maintain biodiversity features in the face of global change (Plumptre et al., 2021).

Spatial analyses of patterns of phylogenetic diversity have identified evolutionary “hotspots” (i.e., regions with high speciation and/or low extinction rates; e.g., Cape region in South Africa) and also important areas for the conservation of unique features (i.e., traits) for several organisms (Daru et al., 2016; González‐Orozco et al., 2015; Thornhill et al., 2017). Studies on biodiversity hotspots can be combined with species distribution modeling to link present and future areas of evolutionary interest under different climate change scenarios (Cai et al., 2021; Qian et al., 2023; Rodriguez et al., 2022; Voskamp et al., 2022), allowing for more data‐driven conservation approaches in the face of global change. As proposed by Brooks et al. (2015), this concept could be included in the definition of Key Biodiversity Areas (KBAs), defined by the IUCN as “sites that contribute significantly to the global persistence of biodiversity”.

Bold application of approaches to enhance evolutionary potential combined with novel techniques offers possibilities to preserve target species and entire ecosystems in the face of anthropogenic stress, by enhancing resilience and mitigating the impacts of disturbance. Two examples are human‐assisted evolution and hybridization‐enhanced evolutionary rescue. Assisted evolution for developing coral stocks with enhanced stress tolerance through the acceleration of naturally occurring processes, e.g., selective breeding to generate certain genotypes exhibiting desirable phenotypic traits, is a promising research (van Oppen et al., 2015). Intraspecific hybridization has been proposed as a conservation management tool, focused on enhancing and preserving the adaptive potential and survival of populations at risk of certain species, in coral reefs, for example (Chan et al., 2019).

## 4 BARRIERS TO HARNESSING THE LINK BETWEEN EVOLUTION AND SUSTAINABILITY

There is strong potential for evolutionary research and insights to contribute to multiple SDGs and add to an improved response to key societal challenges. Yet, there are few cases of effective and explicit implementation of these insights in policies, with exceptions like the genetic management of endangered species, genetic identification of fish stocks, and genomic tracking of the evolution of pathogens. The lack of consideration of evolution is an important barrier towards sustainability (Vázquez‐Domínguez et al., 2024), exemplified by cases like economies driving the impoverishment of genetic diversity in food production and lapses in antibiotic stewardship jeopardizing our future health. Barriers that hamper the implementation of evolutionary insights into policy might be:

1. Perception: While evolution can have important consequences for management, food production, and health, it is often perceived as being irrelevant to practical decision‐making either because, among others, it is seen to operate on very long time scales or has been tainted by historical misapplication of evolution for non‐scientific arguments, promoting ideology and discrimination (Dikötter, 1998). The numerous examples we have highlighted here, where rapid evolution can impact ecosystem services and health, reinforces the need to consider evolution in decision‐making.
2. Time frame: There is often a trade‐off between short‐term and long‐term benefits of policies, yet with long‐term damage, such as with size‐selective fisheries, crop homogenization and loss of crop diversity, and the role of genetics in extinction.
3. Conflicts between individual and collective benefits when, for instance, prevention of the development of antibiotic resistance necessitates a reduction in antibiotic usage, while some patients might benefit from a more liberal usage.

These barriers can be overcome by bringing evolutionary biologists and sustainability researchers together to inform sustainability policy and practices. We must collectively recognize that the foundational processes of evolution responsible for the incredible diversity of life on earth continue to operate. The choice at hand is how and when to apply these principles to ensure we achieve sustainability. The ultimate goal of our perspective is to help foster such transdisciplinary collaboration and implementation.

## 5 CONCLUSIONS

Evolution influences key processes relevant to how populations, communities, and ecosystems respond to environmental change, their resilience to global change, agricultural production, medicine, health, well‐being, and the maintenance of biodiversity. The holistic approach typical of sustainability science needs to take past and contemporary evolution into account, as these processes underpin and sustain sustainable ecosystem services and Nature's Contributions to People, providing options for yet unrevealed applications.

While the integration of evolutionary tools and the application of evolutionary insights in applied fields like food and fiber production, health, and species conservation can be quite straightforward, harnessing the implications of contemporary evolution in the context of global change can be more challenging (Barraclough, 2015). Moreover, not all evolution is adaptive, so recognizing when evolutionary processes can be harnessed to reinforce sustainability remains a major challenge. When it comes to natural populations, however, one recommendation is clear: enhance the maintenance of genetic variation necessary to support evolution (Díaz et al., 2020). From time to time, it may be helpful to supplement natural evolutionary processes in targeted conditions, for instance, for the management of infectious disease and critically endangered wild populations, but most of the resilience fostered by genetic adaptation will have to result from evolution as a natural process. It is crucially important that the Montreal‐Kunming Global Biodiversity Framework (Obura, 2023) recognizes the maintenance of genetic diversity as a goal, stressing the need to monitor this component of biodiversity (Heuertz et al., 2023; Hoban et al., 2022; Robuchon et al., 2023).

Our perspective illustrates how evolutionary data, theory, concepts, insights, and technological toolbox can contribute to the UN Sustainable Development Goals. We hope that it will inspire scientists to reflect on the possibilities to take advantage of genetic and phylogenetic diversity, and on the consequences of decisions and policies on sustainable development for biodiversity. We urge biologists to study how evolutionary biology can harness sustainability measures and policies, for example, by developing better tools and methods for predicting evolutionary outcomes. Further, we encourage societal actors to consider evolutionary insights into planning considerations, management, and policy development as they are key to achieving sustainability in society and of Earth's ecosystem on which we depend.

## CONFLICT OF INTEREST STATEMENT

The authors declare that they do not have any conflict of interest.

## Data Availability

Data sharing is not applicable to this article as no new data were created or analysed.
